# Supplementary material for: Neurotrophic Factors in Experimental Cerebral Acanthamoebiasis
Source: Int J Mol Sci. 2022 Apr 29;23(9):4931. doi: 10.3390/ijms23094931 (PMC9103668; doi:10.3390/ijms23094931)
Supplement: Supplementary file 1 [file ijms-23-04931-s001.zip › ijms-1654907-supplementary.pdf]

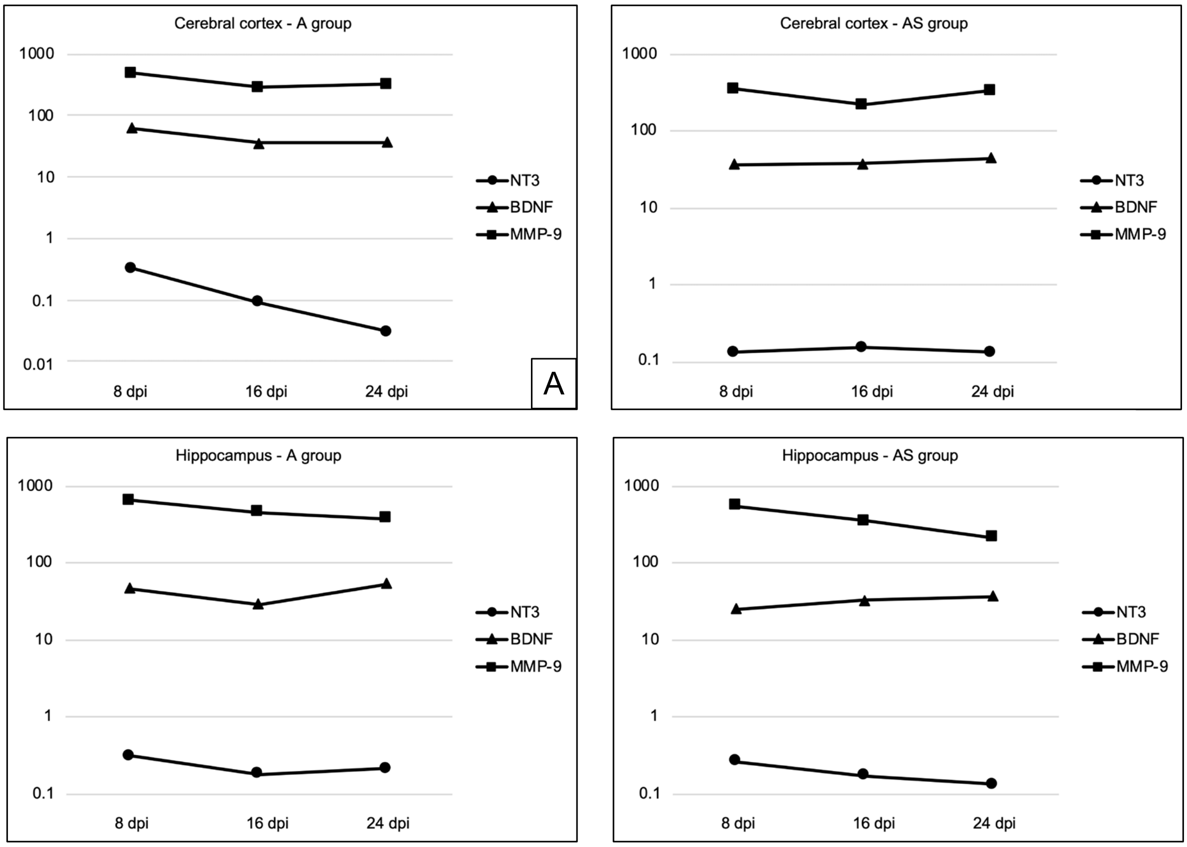

Figure S1. Concentration of neurotrophin 3 (NT3), brain-derived neutrophic factor (BDNF) [present study] and matrix metalloproteinase 9 (MMP-9) [Łanocha-Arendarczyk et al. 2018] in the cerebral cortex of immunocompetent (A) and immunosuppressed mice (B) infected with *Acanthamoeba* spp. as well as in the hippocampus of immunocompetent (C) and immunosuppressed mice (D) infected with *Acanthamoeba* spp. in different days post infection (dpi)
